# Identification of NRF2 Activation as a Prognostic Biomarker in T-Cell Acute Lymphoblastic Leukaemia

**DOI:** 10.3390/ijms241210350

**Published:** 2023-06-19

**Authors:** María Villa-Morales, Laura Pérez-Gómez, Eduardo Pérez-Gómez, Pilar López-Nieva, Pablo Fernández-Navarro, Javier Santos

**Affiliations:** 1 Department of Biology, Universidad Autónoma de Madrid, 28049 Madrid, Spain; laura.perezgomez01@estudiante.uam.es (L.P.-G.); pilar.lopez@cbm.csic.es (P.L.-N.); 2 Department of Genome Dynamics and Function, Centro de Biología Molecular Severo Ochoa (CBMSO), Consejo Superior de Investigaciones Científicas—Universidad Autónoma de Madrid (CSIC-UAM), 28049 Madrid, Spain; 3 Area of Genetics and Genomics, IIS Fundación Jiménez Díaz, 28040 Madrid, Spain; 4 Institute for Molecular Biology-IUBM, Universidad Autónoma de Madrid, 28049 Madrid, Spain; 5 Departamento de Bioquímica y Biología Molecular, Universidad Complutense de Madrid, 28040 Madrid, Spain; eduperez@ucm.es; 6 Instituto de Investigación Hospital 12 de Octubre, 28041 Madrid, Spain; 7 Unit of Cancer and Environmental Epidemiology, Centro Nacional de Epidemiología, Instituto de Salud Carlos III, 28029 Madrid, Spain; pfernandezn@isciii.es; 8 Networking Biomedical Research Centre of Epidemiology and Public Health (CIBERESP), 28029 Madrid, Spain

**Keywords:** T-cell acute lymphoblastic leukaemia (T-ALL), NRF2 (nuclear factor erythroid 2-related factor 2), prognostic biomarker

## Abstract

The standard-of-care treatment of T-cell acute lymphoblastic leukaemia (T-ALL) with chemotherapy usually achieves reasonable rates of initial complete response. However, patients who relapse or do not respond to conventional therapy show dismal outcomes, with cure rates below 10% and limited therapeutic options. To ameliorate the clinical management of these patients, it is urgent to identify biomarkers able to predict their outcomes. In this work, we investigate whether NRF2 activation constitutes a biomarker with prognostic value in T-ALL. Using transcriptomic, genomic, and clinical data, we found that T-ALL patients with high *NFE2L2* levels had shorter overall survival. Our results demonstrate that the PI3K-AKT-mTOR pathway is involved in the oncogenic signalling induced by NRF2 in T-ALL. Furthermore, T-ALL patients with high *NFE2L2* levels displayed genetic programs of drug resistance that may be provided by NRF2-induced biosynthesis of glutathione. Altogether, our results indicate that high levels of *NFE2L2* may be a predictive biomarker of poor treatment response in T-ALL patients, which would explain the poor prognosis associated with these patients. This enhanced understanding of NRF2 biology in T-ALL may allow a more refined stratification of patients and the proposal of targeted therapies, with the ultimate goal of improving the outcome of relapsed/refractory T-ALL patients.

## 1. Introduction

T-cell acute lymphoblastic leukaemia (T-ALL) results from the malignant transformation of T-cell progenitors at various stages of differentiation [1]. The landscape of genetic alterations in T-ALL includes chromosomal abnormalities, such as 9p deletions and translocations affecting the T-cell receptor genes and leading to overexpression of oncogenic transcription factors; additionally, mutations in more than 100 different genes have been identified, most of them at low frequency [2]. Apart from the two most frequently deregulated pathways in T-ALL, which are NOTCH1 and JAK/STAT, hyperactivation of PI3K-AKT-mTOR and RAS-MAPK pathways are also relevant [3]. Hyperactivation of the PI3K-AKT-mTOR pathway in T-ALL is mainly caused by inactivating mutations or deletions of *PTEN*, the main negative regulator of the pathway [2].

Despite the continuous efforts to understand the molecular complexity of T-ALL, treatments are still based on chemotherapeutic regimens that may be followed by hematopoietic stem cell transplantation in high-risk patients [4,5]. Such treatments achieve reasonable rates of initial complete response; however, around 40% of adult and 15% of paediatric T-ALL patients still relapse. Unfortunately, patients who relapse or do not respond to first-line treatment show very poor prognoses as cure rates decrease to 7% [1]. A major problem for T-ALL is the lack of reliable prognostic factors besides minimal residual disease [6]. Thus, to improve the clinical management of these patients, particularly those who are refractory to treatments and those who relapse, it is necessary to identify biomarkers with a prognostic value. Such markers will allow a more refined risk-based stratification of patients which, in consequence, will improve the chances of long-term therapeutic success. To date, the involvement and mechanisms underlying NRF2 deregulation in T-ALL need to be better characterised before it can be proposed as a reliable prognostic biomarker [7].

The transcription factor NRF2, encoded by the *NFE2L2* (nuclear factor erythroid 2-related factor 2) gene, belongs to the Cap’n’Collar (CNC) subfamily of basic leucine zipper (bZIP) transcription factors, which comprises the nuclear factor erythroid-derived 2 (NFE2) and NRF1, NRF2, and NRF3 [8]. NRF2 is one of the main orchestrators of the cellular antioxidant response by inducing the transcription of genes containing antioxidant response elements (AREs) [9] to protect cells from the effects of exogenous and endogenous insults causing oxidative stress [10]. NRF2 can have either beneficial or detrimental functions in cancer cells depending on the tumour stage and signal persistence. In the early stages, transient activation of the antioxidative role of NRF2 seems to be relevant for avoiding premalignant tumorigenesis, DNA damage, and initial cancer mutations [11,12]. However, at advanced stages, persistent NRF2 hyperactivation creates an environment that may favour the survival of tumour cells by protecting them from excessive oxidative stress, chemotherapeutic agents, or radiotherapy [10,13,14]. In cancer, NRF2 can also play a role in metabolic reprogramming, directing metabolic intermediates into the Warburg and pentose phosphate pathways to support proliferative growth and redox homeostasis [8,15,16,17].

Different mechanisms can lead to aberrant sustained activation of NRF2 signalling in tumour cells, including somatic mutations in *NFE2L2* or *KEAP1*, exon 2 skipping in *NFE2L2*, *KEAP1* downregulation due to promoter hypermethylation, and transcriptional activation of *NFE2L2* gene [17]. Recent research has found elevated levels of NRF2 in a number of solid tumour forms, including head and neck, gastric, breast, gallbladder, and ovarian cancer [18,19,20,21,22]. In haematological malignancies, high expression of NRF2 has been found in patients of acute myeloid leukaemia (AML) [23,24,25], chronic lymphocytic leukaemia (CLL) [26] and acute lymphoblastic leukaemia (ALL) [7,27].

Notably, many clinical studies have shown that accumulation of NRF2 is associated with poor prognosis in tumours of the brain, lung, esophageal, breast, hepatocellular, bladder, pancreatic, cervical, melanoma, ovarian, gastric, and colorectal (reviewed in [17]), indicating that NRF2 and its downstream effectors can be considered as prognostic factors in a wide range of cancers. In haematological malignancies, overexpression of NRF2 has been associated with drug resistance and disease progression in AML [23,24,25,28,29,30]. In B-cell acute lymphoblastic leukaemia (B-ALL), overexpression of NRF2 has been directly associated with drug resistance to vincristine [27]. In T-cell acute lymphoblastic leukaemia (T-ALL), increased levels of NRF2 were observed in patients classified as therapy-resistant, according to their positivity in minimal residual disease (MRD), but expression levels largely overlapped between the groups, suggesting that the use of NRF2 levels as a prognostic biomarker in this context would be challenging [7].

In this study, we sought to investigate whether NRF2 activation may constitute a biomarker with prognostic value in T-ALL, able to predict the outcome of the patients; this would be particularly relevant for patients who do not respond to current treatments and for those who relapse and would open new venues for therapeutic intervention.

## 2. Results

### 2.1. High Expression of NFE2L2 Associates with Poor Prognosis in T-Cell Acute Lymphoblastic Leukaemia

**Figure 1:**
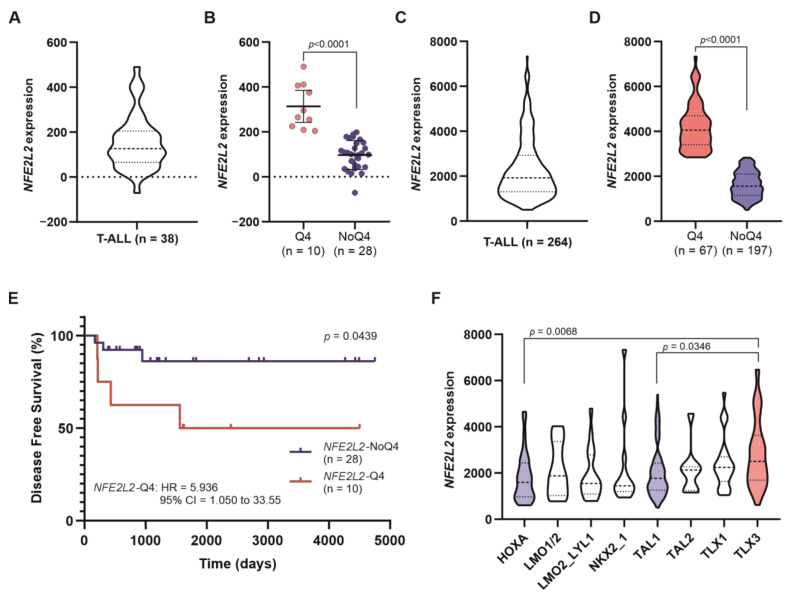
High levels of *NFE2L2* associate with poor prognosis in T-cell acute lymphoblastic leukaemia. (**A**) Violin plot representing *NFE2L2* expression (scaled Affymetrix average difference expression values) in 38 T-ALL patients obtained from the dataset published in Ref. [32]. (**B**) Scatter plot representing *NFE2L2* expression (scaled Affymetrix average difference expression values) in patients belonging or not to the upper quartile of *NFE2L2* expression (Q4 and NoQ4 represented with red and blue dots, respectively). The mean with a 95% confidence interval is shown. Mann–Whitney test was used to determine the *p*-value. (**C**) Violin plot representing *NFE2L2* expression (DESeq2 normalised counts) in 264 patients from the TARGET cohort. (**D**) Violin plots representing *NFE2L2* expression (DESeq2 normalised counts) in patients belonging or not to the upper quartile of *NFE2L2* expression (Q4 and NoQ4, respectively). Mann–Whitney test was used to determine the *p*-value. (**E**) Kaplan–Meier disease-free survival curve analysis in T-ALL patients belonging or not to the upper quartile of *NFE2L2* expression (*NFE2L2*-Q4 and *NFE2L2*-NoQ4, respectively), obtained from the dataset published in Ref. [32]. Log-rank (Mantel-Cox) test was used to determine the *p*-value; HR, hazard ratio (Mantel–Haenszel); 95% CI, and 95% confidence interval of the ratio. (**F**) Violin plots representing *NFE2L2* expression (DESeq2 normalised counts) in patients belonging to the subgroups HOXA (n = 33), LMO1/2 (n = 10), LMO2-LYL1 (n = 18), NKX2-1 (n = 14), TAL1 (n = 87), TAL2 (n = 8), TLX1 (n = 26) and TLX3 (n = 46) (as defined in the Ref. [31]). Krustal-Wallis was used to test for statistical significance. Adjusted *p*-values calculated by Dunn’s test are shown.

Using transcriptional data from a cohort of 38 patients with T-cell acute lymphoblastic leukaemia (T-ALL), we found that *NFE2L2* expression has a variable pattern, with heterogeneous levels of *NFE2L2* (Figure 1A) and a significant difference in *NFE2L2* expression between patients within the upper quartile (Q4) and the rest of patients (NoQ4) (Figure 1B). This was confirmed in a larger cohort of 264 T-ALL (Figure 1C,D). Whole exome sequencing data revealed no somatic mutations affecting the coding sequence of either *NFE2L2* or *KEAP1*. This supports that transcriptional activation of *NFE2L2* may be a relevant mechanism responsible for aberrant activation of NRF2 signalling in T-ALL.

With the use of the available clinical data, we performed a disease-free survival analysis to determine whether elevated *NFE2L2* expression had any implication on the outcome of T-ALL. The results showed that patients within the upper quartile of *NFE2L2* expression levels (*NFE2L2*-Q4) had substantially shorter disease-free survival than the rest of the patients (Figure 1E). We performed additional analyses of the clinic-pathological data associated with the TARGET cohort, for whom long-term survival data are still immature. A multiple comparisons of *NFE2L2* expression revealed a significant variation (*p*-value = 0.0046) in patients classified into eight subgroups based on genetic alterations and deregulated expression of transcription factor genes [31] as HOXA, LMO1/2, LMO2-LYL1, NKX2-1, TAL1, TAL2, TLX1, and TLX3. In particular, we found that *NFE2L2* expression was significantly higher in patients belonging to the TLX3 subgroup in comparison with those defined as TAL1 and HOXA (Figure 1F). Since TLX3 overexpression correlates with a poor outcome (as reviewed in [6]), this finding supports the notion that elevated *NFE2L2* expression may be predictive of poor prognosis in T-cell acute lymphoblastic leukaemia.

### 2.2. T-ALL Patients with High NFE2L2 Expression Exhibit a Genetic Program of NRF2-Induced Transcriptional Targets and Signalling

**Figure 2:**
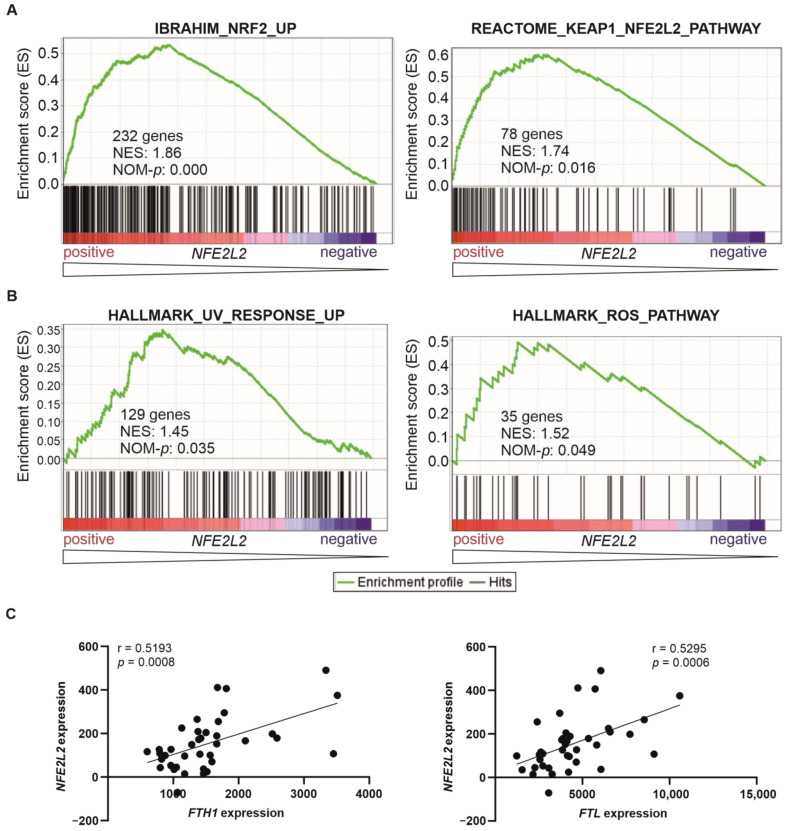
Genetic signatures of NRF2-induced transcriptional targets and signalling correlate positively with *NFE2L2* expression in T-ALL patients. (**A**,**B**) Gene Set Enrichment Analysis was performed in 38 T-ALL patients with publicly available gene expression data [32] based on their *NFE2L2* expression levels as a continuous variable. These signatures were selected from the Molecular Signatures Database (MSigDB), and their systematic names are M42510 (IBRAHIM_NRF2_UP), M45021 (REACTOME_KEAP1_NFE2L2_PATHWAY), M5941 (HALLMARK_UV_RESPONSE_UP), and M5938 (HALLMARK_ REACTIVE_OXYGEN_SPECIES_PATHWAY). The green curve corresponds to the enrichment score (ES) curve, which is the running sum of the weighted score obtained with the GSEA software v4.2.1. ROS, Reactive Oxygen Species; NES, normalised enrichment score; NOM-*p*, nominal *p* value. (**C**) A significant positive correlation between *NFE2L2* and *FTH1* (left) and *FTL* (right) transcriptional levels (scaled Affymetrix average difference expression values) in the cohort from [32]. The values for Spearman r and corresponding *p*-value are shown, although both parametric (Pearson) and non-parametric (Spearman) analyses revealed statistical significance.

In order to determine whether high *NFE2L2* expression leads to increased NRF2 activity in T-ALL patients, we performed Gene Set Enrichment Analysis (GSEA) in the published gene expression dataset [32] of 38 T-ALL patients whose overall survival had been previously analysed. Our results showed that several genetic signatures related to NRF2 activity were significantly enriched in samples from patients exhibiting high *NFE2L2* levels (Figure 2A and Figure S1A). These genetic signatures define the genetic programmes executed by NRF2 in humans, including the consensus set of target genes regulated by NRF2 (IBRAHIM_NRF2_UP) and the transcriptional program promoted by NRF2 (REACTOME_KEAP1_NFE2L2_PATHWAY). This result was validated using transcriptional data from the TARGET cohort (Supplementary Figure S1B).

Consistent with NRF2 best-known role in the induction of the cellular antioxidant response, several genetic signatures related to this function were significantly enriched in samples from patients exhibiting high *NFE2L2* levels (Figure 2B and Figure S1C). We performed a correlation analysis between the expression levels of *NFE2L2* and representative genes related to such antioxidant role; a positive correlation was observed between *NFE2L2* and the expression levels of *FTH1* (Ferritin Heavy Chain 1) and *FTL* (Ferritin Light Chain) (Figure 2C).

### 2.3. Aberrant Activation of the MAPK-ERK and PI3K-AKT-mTOR Signalling Pathways in T-ALL Patients with High NFE2L2

Once determined that T-ALL patients with high *NFE2L2* levels exhibited increased NRF2 signalling, our next step was to investigate the molecular mechanisms activated in these tumours, which may contribute to tumorigenesis.

**Figure 3:**
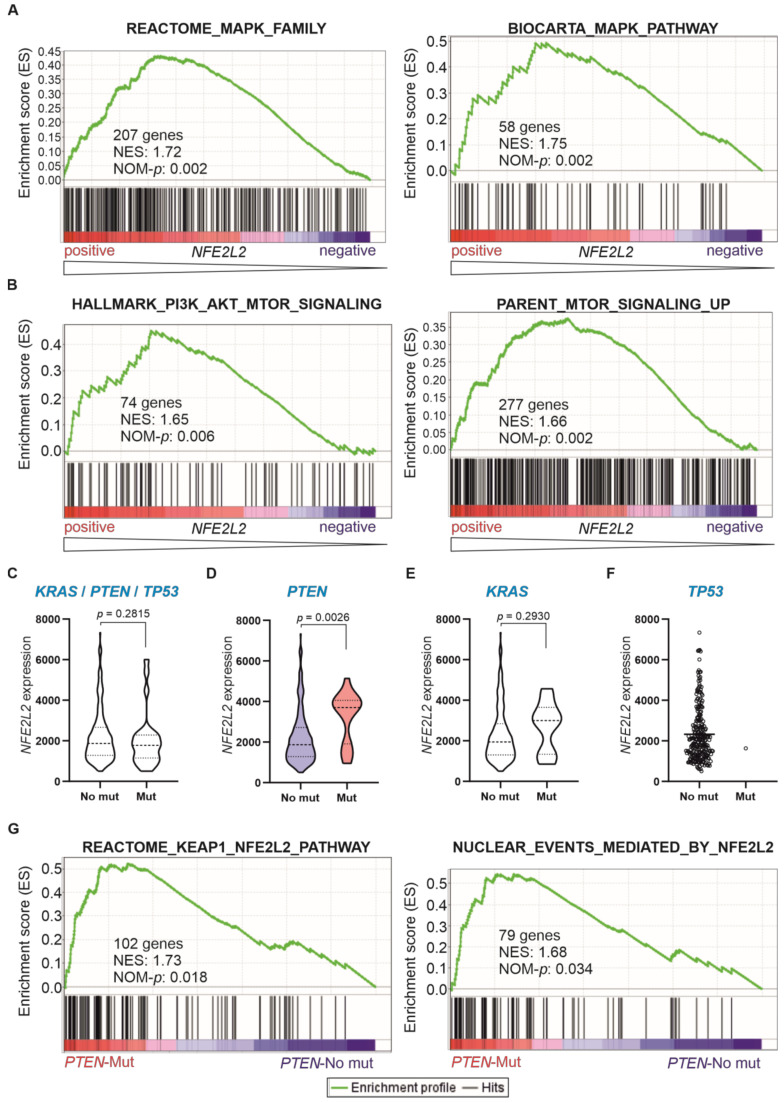
Association between *NFE2L2* expression and MAPK-ERK and PI3K-AKT-mTOR oncogenic signalling in T-ALL. (**A**,**B**) Gene Set Enrichment Analysis (GSEA) was performed in 38 T-ALL patients with publicly available gene expression data [32] based on their *NFE2L2* expression levels as a continuous variable. (**A**) Two representative signatures for MAPK-ERK signalling pathway were selected from the Molecular Signatures Dabatase (MSigDB), and their systematic names are M27565 (REACTOME_MAPK_FAMILY_SIGNALING_CASCADES) and M13863 (BIOCARTA_MAPK_PATHWAY). (**B**) Two representative signatures for PI3K-AKT-mTOR signalling pathway were selected from the Molecular Signatures Dabatase (MSigDB), and their systematic names are M5923 (HALLMARK_PI3K_AKT_MTOR_SIGNALING) and M16909 (PARENT_MTOR_SIGNALING_UP). The green curve corresponds to the enrichment score (ES) curve, which is the running sum of the weighted score obtained with the GSEA software v4.2.1. NES, normalized enrichment score; NOM-*p*, nominal *p* value. (**C**–**F**) Violin or scatter plots representing *NFE2L2* expression (DESeq2 normalized counts) in 249 patients from the TARGET cohort without (No mut) or with (Mut) mutations in *KRAS*, *PTEN* or *TP53*. Mann–Whitney test was used to determine the *p*-value. (**G**) GSEA was performed in 249 T-ALL patients from the TARGET cohort with available data from whole exome sequencing, comparing those with and without mutations in *PTEN* (*PTEN*_Mut vs. *PTEN*_No mut) as discrete phenotypes. These signatures were selected from the Molecular Signatures Dabatase (MSigDB), and their systematic names are M45021 (REACTOME_KEAP1_NFE2L2_PATHWAY) and M45026 (REACTOME_NUCLEAR_EVENTS_MEDIATED_BY_NFE2L2). The green curve corresponds to the enrichment score (ES) curve, which is the running sum of the weighted score obtained with the GSEA software v4.2.1. NES, normalized enrichment score; NOM-*p*, nominal *p* value.

The activity of NRF2 has been associated with the aberrant activation of the MAPK-ERK and PI3K-AKT-mTOR signalling pathways, leading to cancer cell proliferation [26,33,34] and resistance to apoptosis [5]. Accordingly, we observed a significant positive correlation between genetic signatures related to these two signalling pathways and *NFE2L2* expression in T-ALL patients (Figure 3A,B and Supplementary Figure S2A,B).

Oncogenic mutations inducing such proliferative signals, such as activating mutations of *HRAS*/*KRAS* and loss-of-function mutations of *PTEN* or *TP53*, have been reported to stabilise and activate NRF2 [17]. Thus, we aimed to investigate in T-ALL the potential association between the presence of somatic mutations affecting these genes and the aberrant activation of NRF2 signalling. In T-ALL patients from the TARGET cohort, where whole exome sequencing data were available (n = 249), we identified tumour samples bearing oncogenic mutations affecting *KRAS*, *PTEN* or *TP53*. Twenty-five patients exhibited oncogenic mutations affecting any of these genes. From them, only one patient carried a loss-of-function mutation in *TP53*, whereas nine patients exhibited gain-of-function mutations in *KRAS*, and *PTEN* was the most frequently mutated gene, with 17 patients bearing loss-of-function mutations in this gene (Table 1). We compared the expression levels of *NFE2L2* in patients with and without mutations in any of these genes, and we observed no significant difference (Figure 3C). However, when *NFE2L2* expression was assessed in patients exhibiting mutations in these individual genes, we observed that *NFE2L2* levels were significantly higher in patients having loss-of-function mutations in *PTEN* (Figure 3D), which did not occur for *KRAS* or *TP53* (Figure 3E,F). Using a comparison between the groups of patients exhibiting or not loss-of-function mutations in *PTEN* as categorical phenotypes (*PTEN*-mutated vs. *PTEN*-No mutated), we observed a significant enrichment of genetic signatures related to NRF2 activity in the *PTEN*-mutated group (Figure 3G). To determine the degree of the contribution that the *PTEN*-mutated group has in the positive correlation previously observed between PI3K-AKT-mTOR genetic signatures and *NFE2L2* expression in T-ALL, we investigated the enrichment of this pathway only in those cases not exhibiting *PTEN* loss-of-function mutations. A significant positive correlation with *NFE2L2* expression was observed (Supplementary Figure S3). This reinforces the notion that the PI3K-AKT-mTOR pathway is involved in the oncogenic signalling induced by NRF2 in T-ALL, independently of the mechanism responsible for PI3K-AKT-mTOR activation.

### 2.4. T-ALL Patients with High NFE2L2 Levels May Develop a Phenotype of Drug Resistance Provided by NRF2-Induced Biosynthesis of Glutathione

**Figure 4:**
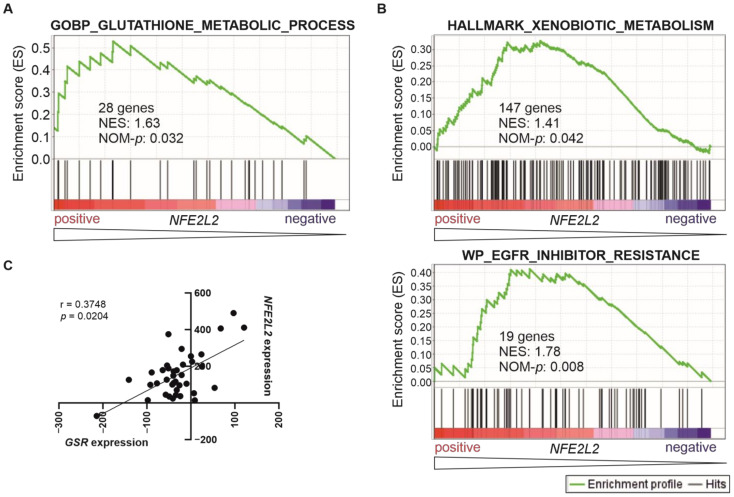
Genetic signatures of glutathione metabolism and drug resistance correlate positively with *NFE2L2* expression in T-ALL patients. (**A**,**B**) Gene Set Enrichment Analysis was performed in 38 T-ALL patients with publicly available gene expression data [32], based on their *NFE2L2* expression levels as a continuous variable. These signatures were selected from the Molecular Signatures Dabatase (MSigDB), and their systematic names are M14708 (GOBP_GLUTATHIONE_METABOLIC_PROCESS), M5934 (HALLMARK_XENOBIOTIC_METABOLISM) and M39839 (WP_EGFR_TYROSINE_KINASE_INHIBITOR_RESISTANCE). The green curve corresponds to the enrichment score (ES) curve, which is the running sum of the weighted score obtained with the GSEA software v4.2.1. NES, normalised enrichment score; NOM-*p*, nominal *p*-value. (**C**) A significant positive correlation between *NFE2L2* and *GSR* transcriptional levels (scaled Affymetrix average difference expression values) in the cohort from [32]. The values for Spearman r and corresponding *p*-value are shown, although both parametric (Pearson) and non-parametric (Spearman) analyses revealed statistical significance.

NRF2 stimulates glutathione (GSH) biosynthesis by driving expression of the GSH biosynthetic genes [36]. The GSH system, similarly to other antioxidant systems, is able to remove reactive oxygen species (ROS) from leukaemia cells [37], protecting the tumour cell from oxidative stress. In line with this, we found a significant positive correlation between a genetic signature related to glutathione metabolism and *NFE2L2* expression in T-ALL patients (Figure 4A and Figure S4A).

NRF2-induced glutathione biosynthesis is the proposed mechanism for drug resistance in NRF2-activated tumour cells. Notably, T-ALL patients with high *NFE2L2* levels showed significant enrichment of genetic signatures related to the metabolism of xenobiotics and drug resistance (Figure 4B and Figure S4B). A positive correlation was found between the expression levels of *NFE2L2* and *GSR* (Glutathione-Disulfide Reductase) when we conducted a correlation analysis between the expression levels of *NFE2L2* and representative genes involved in NRF2-induced xenobiotic metabolism (Figure 4C).

## 3. Discussion

Previous evidence showed that *NFE2L2* expression is indicative of poor prognosis in many cancer types (reviewed in [17]). In T-ALL, previous data were supportive of an oncogenic role for NRF2 [7,38,39]. However, the scarcity of longitudinal specimens hindered definitive conclusions regarding the prognostic value of NRF2 in T-ALL. In this study, the use of a cohort of 38 T-ALL with mature survival data has allowed us to reveal that patients with high *NFE2L2* expression and signal activation presented a significantly reduced disease-free survival compared to those with low *NFE2L2* expression and signalling. In line with this, the analysis of clinic-pathological parameters in the much larger TARGET cohort, consisting of 264 patients with T-ALL, revealed an additional correlation between *NFE2L2* expression and the TLX3 molecular subgroup, which is a reported poor outcome factor (reviewed in [6]). Altogether, these results strongly support that high *NFE2L2* expression is indicative of poor prognosis in T-ALL.

In human cancer, different mechanisms can induce the activation of NRF2 signalling. Somatic mutations of *NFE2L2* and *KEAP1* are one of the main causes of constitutive NRF2 activation, but their frequency varies, importantly, between tumour types. Thus, these genes are mutated in 10–30% of lung cancer, whereas no mutations have been found in *NFE2L2* or *KEAP1* in pancreatic cancer [17]. In a recent study with B-ALL patients, no missense mutations were found in *NFE2L2*, whereas 13 of 30 patients (43%) presented missense mutations affecting *KEAP1* [40]. In the present work, the analysis of whole exome sequencing data from 249 T-ALL patients did not reveal the occurrence of any mutation affecting *NFE2L2* or *KEAP1*. Nevertheless, we confirmed that *NFE2L2* expression is above the mean in around 40% of T-ALL patients from two different cohorts and that it is significantly higher in patients belonging to the upper quartile (*NFE2L2*-Q4), suggesting that an increase in *NFE2L2* transcription would be a key mechanism able to induce activation of NRF2 signalling in T-ALL.

However, the mere transcriptional expression of *NFE2L2* does not ensure its function. NRF2 protein levels are strictly regulated in the cell since NRF2 is constantly poly-ubiquitinated by the CUL3-KEAP1 E3 ubiquitin ligase complex and subjected to degradation by proteasome [10]. Furthermore, NRF2 can also be ubiquitinated by the CUL1-bTrCP complex after being phosphorylated by GSK3 [41,42]. Only upon oxidative and/or electrophilic stress the CUL3-KEAP1 complex is inactivated, and NRF2 is stabilised. NRF2 then can translocate to the nucleus to induce a battery of cytoprotective genes by binding to the antioxidant response element (ARE) on their promoters [9]. Our results demonstrate that high *NFE2L2* expression in T-ALL was accompanied by the implementation of a specific gene expression program leading to NRF2-induced signalling in tumour cells.

The link between high *NFE2L2* expression in cancer cells and the tumorigenesis process itself seems to involve oncogenic pathways, such as MAPK-ERK and PI3K-AKT-mTOR, whose aberrant activation has been strongly associated with the activity of NRF2 [33,34]. Actually, NRF2 has been found to be involved in the proliferation of leukaemia cells [26,34]. In this study, we corroborate that T-ALL patients with high *NFE2L2* levels exhibit genetic programs specifically related to MAPK-ERK and PI3K-AKT-mTOR signalling pathways. The molecular mechanisms behind this have been postulated for several cell and cancer types. For example, in pancreatic and lung cancer, it has been reported that oncogenic KRAS mutants stimulate the transcription of NRF2 via JUN and MYC [43]. However, in this work, we find that *NFE2L2* expression does not differ between T-ALL patients carrying or not *KRAS* oncogenic mutations. Loss-of-function mutations of *TP53* have also been associated with NRF2 aberrant activation since mutant p53 can upregulate *NFE2L2* transcription [44]. However, *TP53* oncogenic mutations were extremely rare among the T-ALL patients whose mutational landscape could be studied in this work and were not associated with any particular increase in *NFE2L2* levels. In cells with active PI3K-AKT-mTOR signalling, GSK3 is phosphorylated and inactivated, and CUL1-bTrCP complex-mediated degradation of NRF2 is impaired [41,42,45]. In hepatocytes, *PTEN* deficiency leading to PI3K-AKT-mTOR activation, combined with *KEAP1* deficiency, induces massive accumulation of NRF2 and NRF2-dependent proliferation [46]. In the present work, we find that T-ALL patients carrying loss-of-function mutations in *PTEN* exhibit increased *NFE2L2* levels and display genetic signatures specified by NRF2 activity. It has been postulated that cancer cells with NRF2 activation may develop “NRF2 addiction”, whereby NRF2 acts as a facultative cancer driver, able to confer malignant phenotypes but only if certain prerequisites of active oncogenic signalling are present in the tumour cell; these prerequisites are not fully unveiled, as they seem to be cell-type specific [17]. Our results in this work point to the involvement of PI3K-AKT-mTOR activation, which in some cases is induced by *PTEN* loss, as a prerequisite for NRF2 participation in T-ALL.

In this scenario of oncogenic PI3K-AKT-mTOR signalling and NRF2 activation, tumour cells can increase antioxidant systems, GSH in particular, to mitigate oxidative stress, which in turn results in drug resistance [26,47]. Numerous pieces of evidence support this notion in haematological malignancies. In AML cell lines, NRF2 activation induces resistance to chemotherapeutic agents [29,30]. In ALL, AML, and CLL patient samples, elevated GSH levels have been associated with anticancer drug resistance [48,49,50,51]. In B-ALL, *NFE2L2* overexpression hinders tumour sensitivity to vincristine, and this occurs through the PI3K-AKT-mTOR pathway [27]. In line with all this, we observe in this work that T-ALL patients with high *NFE2L2* levels display genetic programs of glutathione metabolism and drug resistance. Importantly, T-ALL patients with high *NFE2L2* levels exhibited reduced overall survival, so one might speculate that drug resistance induced by increased GSH could be at least a contributing factor in these tumours. Therefore, high *NFE2L2* levels may be a predictive biomarker of poor treatment response in T-ALL patients.

Future experiments are needed in order to disentangle the molecular details of NRF2 participation in drug sensitivity in T-ALL. In B-ALL, inhibition of NRF2 with brusatol sensitises tumour cells to vincristine [27]. In T-ALL cell lines, brusatol promotes a decrease in cell metabolism and induces apoptosis [38]. Our enhanced understanding of NRF2 biology in T-ALL may allow us to propose targeted therapeutic approaches, which could be used in combination with standard chemotherapy in order to improve the outcome of relapsed/refractory T-ALL patients.

## 4. Materials and Methods

### 4.1. Description of Patient Cohorts

A cohort of 38 T-ALL patients was described in [32]. Samples of cryopreserved lymphoblasts from 59 children and young adults with T-ALL, treated in Total Therapy studies XI–XIII at St. Jude Children’s Research Hospital, were obtained with informed consent at the time of diagnosis before any chemotherapy was given. In our experimental setting, we used clinical data and microarray expression data that were available for 38 of these patients.

A cohort of 264 T-ALL patients was available in the Therapeutically Applicable Research to Generate Effective Treatments (TARGET) initiative. Samples of cryopreserved lymphoblasts from 264 children with T-ALL, treated with Children’s Oncology Group (COG) protocol AALL0434, were collected at diagnosis. Nucleic acid samples were extracted from peripheral blood or bone marrow. In our experimental setting, we used clinical and whole transcriptomic (RNA-seq) data available on all 264 patients and whole exome sequencing (WES) data available in 249 patients. This work is, in part, based upon data generated by the Therapeutically Applicable Research to Generate Effective Treatments (TARGET) initiative, accession number phs000218 and substudy specific accession number phs000464.v19.p8 (TARGET Acute Lymphoblastic Leukemia (ALL) Expansion Phase 2), managed by the National Cancer Institute (NCI). Data for this analysis are accessible through the genotypes and phenotypes database (dbGaP, https://www.ncbi.nlm.nih.gov/projects/gap/cgi-bin/study (accessed on 2 February 2022)). Information about TARGET can be found at http://ocg.cancer.gov/programs/target (accessed on 2 February 2022). The use of data for this cohort requires dbGaP Authorized Access. Access is granted to our group. Thus, omics and associated clinical data are fully available.

### 4.2. In Silico Analysis of Published Datasets

Human gene mRNA expressions in T-ALL were obtained from a microarray dataset published in Ref. [32]. Data files include expression values for 7129 probe sets from the Affymetrix Hu6800FL chip, scaled as described in [32]. Clinical data concerning disease-free survival were also available for this cohort.

Whole transcriptomic (RNA-seq) and clinical data from 264 patients of T-ALL (TARGET cohort) were also available for in silico analysis. Whole exome sequencing (WES) data were available from 249 patients of T-ALL (TARGET cohort); mutations affecting *NFE2L2*, *KEAP1*, *KRAS*, *PTEN*, and *TP53* genes were searched within these data.

### 4.3. Gene Set Enrichment Analysis (GSEA)

Gene set enrichment analysis was performed using the GSEA desktop application from the Broad Institute (v4.2.1) and following instructions in the user guide [52,53]. Enrichment was investigated using *NFE2L2* expression as a continuous variable, which defines a continuous distribution of cases from lowest to highest *NFE2L2* expression in the cohort. In this mode, the GSEA desktop application computes the Pearson correlation of the gene selected as the gene of interest (*NFE2L2* in this case)—using that as a phenotype vector—and each gene in the dataset across all samples. The dataset is assessed for positive and negative correlations with the phenotype vector.

Where indicated, the analysis compared discrete phenotypes defined by categorical labels. To corroborate the enrichment results obtained using *NFE2L2* expression as a continuous variable, we compared the group of patients within the upper quartile of *NFE2L2* expression (*NFE2L2*-Q4) with the rest of the patients in the cohort (*NFE2L2*-NoQ4) (Supplementary Figures S1A,C, S2 and S3A,B).

In addition, we compared discrete phenotypes defined by a group of patients from the TARGET cohort exhibiting loss-of-function mutations in *PTEN* with those not exhibiting them (*PTEN*-mutated vs. *PTEN*-No mutated).

Analyses were run using default parameters (1000 permutations, phenotype permutation and a False Discovery Rate (FDR) cut-off of 25%); a Pearson metric for ranking genes was used for continuous phenotype labels, as recommended. The normalised enrichment score (NES) and nominal *p*-value were measured.

A complete list of statistically significant gene sets identified by GSEA is included in Supplementary Table S1.

### 4.4. Statistical Analysis

Kaplan–Meier curves with disease-free survival, according to *NFE2L2* expression, were generated by using GraphPad Prism v8 (GraphPad Software Inc., La Jolla, CA, USA) with public data available in Ref. [32]. Best threshold cut-offs were selected automatically by the program. Log-rank (Mantel-Cox) test was used to determine the *p*-value; Mantel–Haenszel test was used to determine the hazard ratio.

Statistical analyses were performed with GraphPad Prism v8. The Shapiro–Wilk and Kolmogorov–Smirnov tests were used to check the normality of data groups, and the Levene test was used to check the homogeneity of variances. The differences between independent samples were analysed using the nonparametric Mann–Whitney and Kolmogorov–Smirnov tests for variables not adjusted to normality. Multiple comparisons were performed by Krustal–Wallis analysis, followed by Dunn’s test. The correlation of data was determined by the Spearman test. All *p*-values less than 0.05 were considered statistically significant.

## Figures and Tables

**Table 1:** List of mutations affecting *KRAS*, *PTEN* or *TP53* in T-ALL patients. Non-synonymous mutations affecting the coding sequence of these genes in coding principal transcripts and predicted to be damaging by at least two of three predictors (Polyphen, CONDEL and SIFT) were selected from whole exome sequencing data available from 249 patients.

Patient	Gene *	Mutation	Location	Variation Type	Consequence	HGVS **_cDNA	HGVS **_Protein
1	*PTEN*	-/CT	10:89,717,712	insertion	frameshift_variant	c.737_738insTC	p.Leu247ArgfsTer10
2	*PTEN*	G/T	10:89,692,905	SNV	missense_variant	c.389G>T	p.Arg130Leu
3	*PTEN*	-/G	10:89,711,893	insertion	frameshift_variant	c.510_511insG	p.Gln171AlafsTer9
	*PTEN*	-/CTCACTC	10:89,711,895	insertion	frameshift_variant	c.512_513insCTCACTC	p.Gln171HisfsTer11
4	*KRAS*	C/T	12:25,398,284	SNV	missense_variant	c.35G>A	p.Gly12Asp
5	*KRAS*	C/A	12:25,398,284	SNV	missense_variant	c.35G>T	p.Gly12Val
6	*PTEN*	-/A	10:89,717,671	insertion	frameshift_variant	c.696dup	p.Arg233ThrfsTer10
7	*KRAS*	C/G	12:25,398,284	SNV	missense_variant	c.35G>C	p.Gly12Ala
8	*PTEN*	-/G	10:89,717,676	insertion	frameshift_variant	c.703dup	p.Glu235GlyfsTer8
9	*PTEN*	C/T	10:89,720,741	SNV	stop_gained	c.892C>T	p.Gln298Ter
10	*PTEN*	T/A	10:89,717,739	SNV	missense_variant	c.764T>A	p.Val255Glu
11	*PTEN*	-/G	10:89,717,699	insertion	frameshift_variant	c.724dup	p.Glu242GlyfsTer11
12	*PTEN*	A/G	10:89,711,903	SNV	missense_variant	c.521A>G	p.Tyr174Cys
	*PTEN*	-/G	10:89,717,672	insertion	frameshift_variant	c.696_697insG	p.Arg233AlafsTer10
	*PTEN*	C/T	10:89,717,672	SNV	stop_gained	c.697C>T	p.Arg233Ter
13	*PTEN*	-/ CAGCCGCCGCTTTTGGAGGG	10:89,717,672	insertion	frameshift_variant	c.697_ 698insAGCCGCCGCTTTTGGAGGGC	p.Arg233GlnfsTer30
14	*KRAS*	C/A	12:25,398,284	SNV	missense_variant	c.35G>T	p.Gly12Val
15	*PTEN*	-/GGTGT	10:89,624,297	insertion	frameshift_variant	c.70_71insGGTGT	p.Asp24GlyfsTer4
	*PTEN*	-/GT	10:89,624,298	insertion	frameshift_variant	c.71_72insGT	p.Asp24GlufsTer3
16	*PTEN*	-/T	10:89,717,672	insertion	frameshift_variant	c.696_697insT	p.Arg233SerfsTer10
	*PTEN*	C/T	10:89,717,672	SNV	stop_gained	c.697C>T	p.Arg233Ter
17	*KRAS*	C/T	12:25,398,281	SNV	missense_variant	c.38G>A	p.Gly13Asp
18	*PTEN*	C/T	10:89,692,904	SNV	stop_gained	c.388C>T	p.Arg130Ter
	*PTEN*	C/T	10:89,720,852	SNV	stop_gained	c.1003C>T	p.Arg335Ter
19	*KRAS*	G/C	12:25,398,218	SNV	missense_variant	c.101C>G	p.Pro34Arg
20	*KRAS*	C/T	12:25,398,284	SNV	missense_variant	c.35G>A	p.Gly12Asp
	*PTEN*	-/AA	10:89,692,954	insertion	frameshift_variant	c.439_440dup	p.Ala148ArgfsTer6
21	*PTEN*	C/T	10:89,692,805	SNV	stop_gained	c.289C>T	p.Gln97Ter
22	*KRAS*	C/T	12:25,398,284	SNV	missense_variant	c.35G>A	p.Gly12Asp
	*PTEN*	A/T	10:89,717,636	SNV	stop_gained	c.661A>T	p.Lys221Ter
23	*PTEN*	ATAG/-	10:89,725,147	deletion	frameshift_variant	c.1133_1136del	p.Arg378IlefsTer37
24	*TP53*	G/A	17:7,577,094	SNV	missense_variant	c.844C>T	p.Arg282Trp
	*TP53*	G/A	17:7,577,094	SNV	missense_variant	c.844C>T	p.Arg282Trp
25	*KRAS*	C/A	12:25,398,284	SNV	missense_variant	c.35G>T	p.Gly12Val

* Names of the genes according to HUGO Gene Nomenclature Committee (HGNC). ** Sequence variants at the RNA (cDNA) and protein level according to HGVS nomenclature, following the recommendations in [35].

## Data Availability

This work, in part, is based upon data generated by the Therapeutically Applicable Research to Generate Effective Treatments (TARGET) initiative, accession number phs000218 and substudy specific accession number phs000464.v19.p8 (TARGET Acute Lymphoblastic Leukemia (ALL) Expansion Phase 2), managed by the National Cancer Institute (NCI). Data for this analysis are accessible through the genotypes and phenotypes database (dbGaP, https://www.ncbi.nlm.nih.gov/projects/gap/cgi-bin/study (accessed on 2 February 2022)). Information about TARGET can be found at http://ocg.cancer.gov/programs/target (accessed on 2 February 2022). The use of data for this cohort requires dbGaP Authorized Access. Access is granted to our group; thus, omics and associated clinical data are fully available.

## Supplementary Materials

The following supporting information can be downloaded at: https://www.mdpi.com/article/10.3390/ijms241210350/s1.
