# Supplementary material for: Identification of NRF2 Activation as a Prognostic Biomarker in T-Cell Acute Lymphoblastic Leukaemia
Source: Int J Mol Sci. 2023 Jun 19;24(12):10350. doi: 10.3390/ijms241210350 (PMC10299336; doi:10.3390/ijms241210350)
Supplement: Supplementary file 1 [file ijms-24-10350-s001.zip › Supplementary Data_R_MVM.pdf]

SUPPLEMENTARY TABLES AND FIGURES

Supplementary Table S1. List of statistically significant gene sets identified by Gene Set Enrichment Analysis (GSEA). Details on the patients and type of analysis, the number of genes (size) of each gene set and the enrichment values are indicated. ES, enrichment score. NES, normalized enrichment score. NOM, nominal. FDR, false discovery rate.

COHORT	TYPE OF ANALYSIS	BIOLOGICAL PROCESS	STANDARD NAME IN MSigDB	SIZE	ES	NES	NOM p-val	FDR q-val
**T-ALL cohort described in [32]**	***NFE2L2* expression as a continuous variable**	NRF2-signalling	IBRAHIM_NRF2_UP	232	0.53317404	1.8626792	0.0	0.0047281324
			WP_NRF2ARE_REGULATION	19	0.5735497	1.6477796	0.00984252	0.024935974
			REACTOME_KEAP1_NFE2L2_PATHWAY	78	0.5981252	1.7403532	0.015841585	0.016597716
			REACTOME_NUCLEAR_EVENTS_MEDIATED_BY_NFE2L2	65	0.6006875	1.7091892	0.02385686	0.017106641
		"HALLMARKS" module (MSigDB)	HALLMARK_PROTEIN_SECRETION	62	0.5653247	1.8345639	0.0	0.09958092
			HALLMARK_PI3K_AKT_MTOR_SIGNALING	74	0.45077482	1.6486194	0.00589391	0.18221839
			HALLMARK_APOPTOSIS	132	0.44342795	1.7128903	0.008048289	0.15528475
			HALLMARK_P53_PATHWAY	107	0.3945797	1.5899252	0.014583333	0.1719766
			HALLMARK_COMPLEMENT	151	0.37861565	1.5461987	0.02964427	0.14310727
			HALLMARK_UV_RESPONSE_UP	129	0.34755042	1.4482782	0.034862384	0.15014628
			HALLMARK_ANDROGEN_RESPONSE	63	0.38719532	1.4868444	0.036190476	0.12407464
			HALLMARK_ALLOGRAFT_REJECTION	163	0.42897388	1.6484371	0.036437247	0.1366638
			HALLMARK_XENOBIOTIC_METABOLISM	147	0.32749376	1.4100819	0.042168673	0.1400223
			HALLMARK_HYPOXIA	133	0.34169656	1.4197465	0.042990655	0.1570905
			HALLMARK_IL2_STAT5_SIGNALING	128	0.3887383	1.5210602	0.04347826	0.12652451
			HALLMARK_MTORC1_SIGNALING	139	0.465061	1.5441027	0.049079753	0.12886308
			HALLMARK_REACTIVE_OXYGEN_SPECIES_PATHWAY	35	0.49109906	1.5158067	0.049335863	0.1209114
		"PI3K-AKT-MTOR" and "MAPK" signatures selected from "C2" module (MSigDB)	PARENT_MTOR_SIGNALING_UP	277	0.37444335	1.6406112	0.0020325202	0.007790798
			KEGG_MTOR_SIGNALING_PATHWAY	28	0.5441033	1.7170228	0.0038240917	0.0047743064
			BIOCARTA_MAPK_PATHWAY	58	0.49348474	1.7219363	0.003992016	0.0071614594
			REACTOME_MAPK_FAMILY_SIGNALING_CASCADES	207	0.43142	1.682271	0.005988024	0.007134331
			KEGG_MAPK_SIGNALING_PATHWAY	168	0.3319397	1.4616756	0.01192843	0.025840156
		"Glutathione" signatures selected from MSigDB	GOBP_GLUTATHIONE_METABOLIC_PROCESS	28	0.52879226	1.632531	0.031578947	0.15759353
		"Drug resistance" signatures (MSigDB)	TOOKER_GEMCITABINE_RESISTANCE_DN	73	0.4613966	1.6166724	0.007889546	0.13441806
			WP_EGFR_TYROSINE_KINASE_INHIBITOR_RESISTANCE	64	0.41430157	1.5669923	0.01778656	0.12601322
			TOOKER_GEMCITABINE_RESISTANCE_UP	50	0.5300307	1.7250205	0.022177419	0.12857738
	***NFE2L2*-Q4 *vs* *NFE2L2*-NoQ4**	NRF2-signalling	IBRAHIM_NRF2_UP	232	0.5104008	1.8863534	0.0021598272	0.004589963
			BIOCARTA_ARENRF2_PATHWAY	15	0.5700337	1.6469202	0.010638298	0.026081197
			REACTOME_KEAP1_NFE2L2_PATHWAY	78	0.5533545	1.6898936	0.02455357	0.03298653
			WP_NRF2ARE_REGULATION	19	0.5487138	1.6037898	0.028747434	0.0284945
			REACTOME_NUCLEAR_EVENTS_MEDIATED_BY_NFE2L2	65	0.5592376	1.6717248	0.03539823	0.029416563
		"Reactive oxygen species" signatures selected from "C2" module (MSigDB)	GOBP_REGULATION_OF_REACTIVE_OXYGEN_SPECIES_METABOLIC_PROCESS	94	0.4241613	1.6679244	0.009195402	0.028278569
			GOBP_REACTIVE_OXYGEN_SPECIES_BIOSYNTHETIC_PROCESS	33	0.48617157	1.6144937	0.011111111	0.03406597
			GOBP_REGULATION_OF_REACTIVE_OXYGEN_SPECIES_BIOSYNTHETIC_PROCESS	25	0.54969966	1.706309	0.013215859	0.04088131
			GOBP_POSITIVE_REGULATION_OF_REACTIVE_OXYGEN_SPECIES_METABOLIC_PROCESS	48	0.45550522	1.5888894	0.015452539	0.031796668
			GOBP_CELLULAR_RESPONSE_TO_REACTIVE_OXYGEN_SPECIES	89	0.3539509	1.4577864	0.021881837	0.05894144
			REACTOME_DETOXIFICATION_OF_REACTIVE_OXYGEN_SPECIES	25	0.5179814	1.5627236	0.02631579	0.030678552
			GOBP_REACTIVE_OXYGEN_SPECIES_METABOLIC_PROCESS	141	0.34130841	1.4227666	0.046511628	0.057516515
		"PI3K-AKT-MTOR" and "MAPK" signatures selected from "C2" module (MSigDB)	PARENT_MTOR_SIGNALING_UP	277	0.3938268	1.7989123	0.0	0.015922444
			REACTOME_MAPK_FAMILY_SIGNALING_CASCADES	207	0.4126897	1.6954294	0.006756757	0.039081443
			REACTOME_ONCOGENIC_MAPK_SIGNALING	47	0.44746003	1.5985909	0.01091703	0.02773023
			BIOCARTA_MAPK_PATHWAY	58	0.4388933	1.5999947	0.030237582	0.0409535
			HALLMARK_PI3K_AKT_MTOR_SIGNALING	74	0.3950069	1.5217075	0.034802783	0.10181297
			KEGG_MAPK_SIGNALING_PATHWAY	168	0.30408698	1.3644867	0.035799522	0.10343849
			WP_MAPK_SIGNALING_PATHWAY	159	0.31780806	1.3835868	0.040572792	0.11654138
		"Glutathione" signatures selected from MSigDB	GOBP_GLUTATHIONE_METABOLIC_PROCESS	28	0.48860592	1.5488015	0.036	0.0859133
		"Drug resistance" signatures (MSigDB)	TOOKER_GEMCITABINE_RESISTANCE_DN	73	0.44713596	1.6085708	0.016161617	0.12756611
			KESHELAVA_MULTIPLE_DRUG_RESISTANCE	31	0.4836802	1.6323656	0.023809524	0.22686258
**T-ALL cohort from TARGET**	***NFE2L2* expression as a continuous**	NRF2-signalling	IBRAHIM_NRF2_UP	529	0.45389324	1.756569	0.024475524	0.056212034
			IBRAHIM_NRF2_DOWN	160	-0.46884874	-1.7939197	0.008658009	0.035859738
	***PTEN*-mutated *vs* *PTEN*-No mutated**	NRF2-signalling	REACTOME_KEAP1_NFE2L2_PATHWAY	102	0.51239353	1.6889881	0.031512607	0.047934383
			REACTOME_NUCLEAR_EVENTS_MEDIATED_BY_NFE2L2	79	0.53290725	1.662422	0.044989776	0.02736938
	***NFE2L2* expression as a continuous variable in *PTEN*-No mutated**	"PI3K-AKT-MTOR" signatures selected from "C2" module (MSigDB)	REACTOME_PI3K_CASCADE	68	0.40828627	1.5837905	0.019264448	0.11048066

Supplementary Figure S1

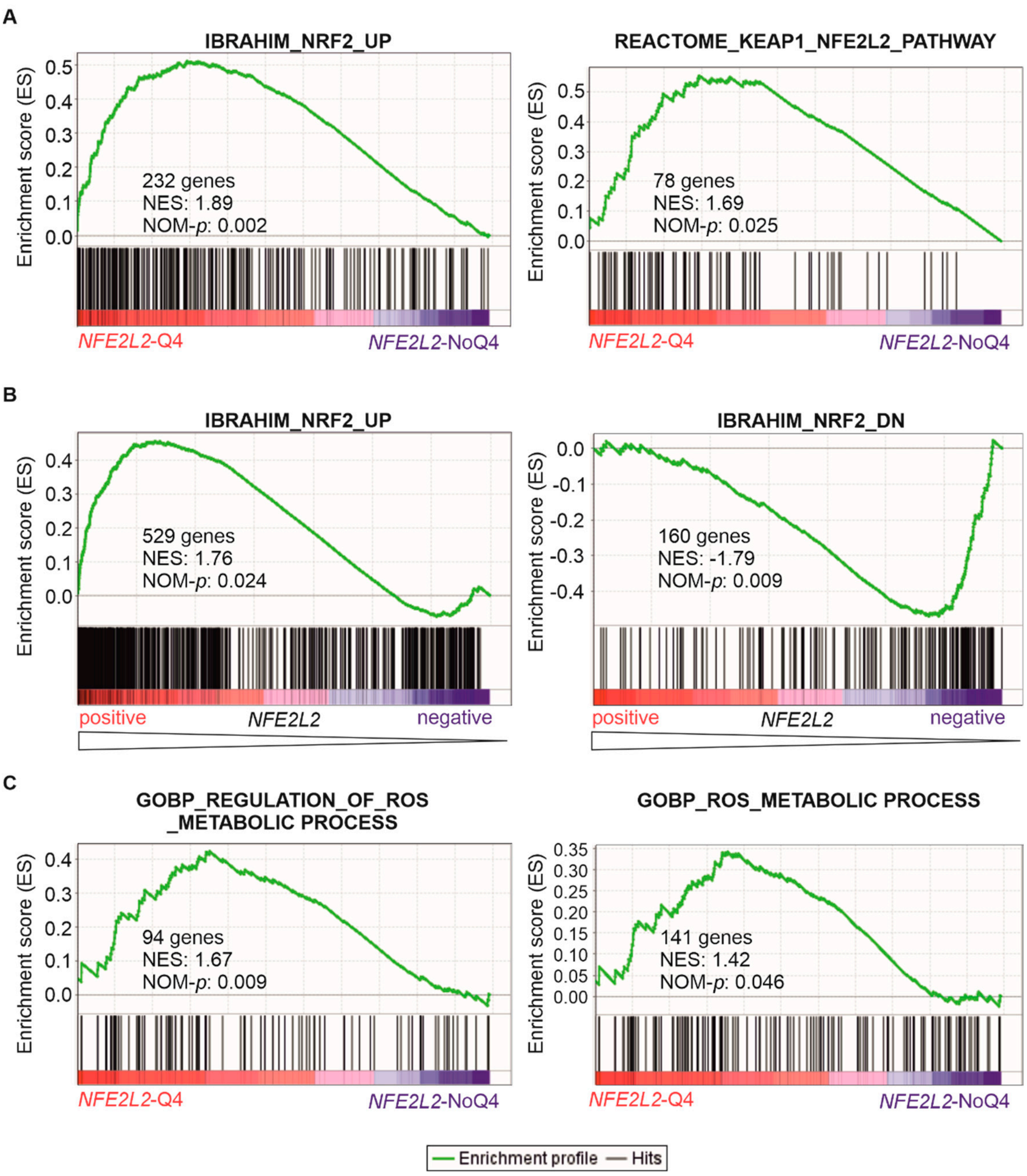

Supplementary Figure S1. Genetic signatures of NRF2-induced transcriptional targets and signalling in T-ALL patients with high *NFE2L2* expression. (A, C) Gene Set Enrichment Analysis (GSEA) was performed in 38 T-ALL patients with publicly available gene expression data [32], comparing patients belonging or not to the upper quartile of *NFE2L2* expression (*NFE2L2*-Q4 *vs* *NFE2L2*-NoQ4). **(B)** GSEA was performed in 264 T-ALL patients (TARGET cohort), based on their *NFE2L2* expression levels as a continuous variable. These signatures were selected from the Molecular Signatures Dabatase (MSigDB), and their systematic names are M42510 (IBRAHIM_NRF2_UP), M45021 (REACTOME_KEAP1_NFE2L2_PATHWAY), M42513 (IBRAHIM_NRF2_DOWN), M13446 (GOBP_REGULATION_OF_REACTIVE_OXYGEN_SPECIES_METABOLIC_PROCESS) and M15990 (GOBP_REACTIVE_OXYGEN_SPECIES_METABOLIC_PROCESS). The green curve corresponds to the enrichment score (ES) curve, which is the running sum of the weighted score obtained with the GSEA software v4.2.1. ROS, Reactive Oxygen Species; NES, normalized enrichment score; NOM-*p*, nominal *p* value.

Supplementary Figure S2

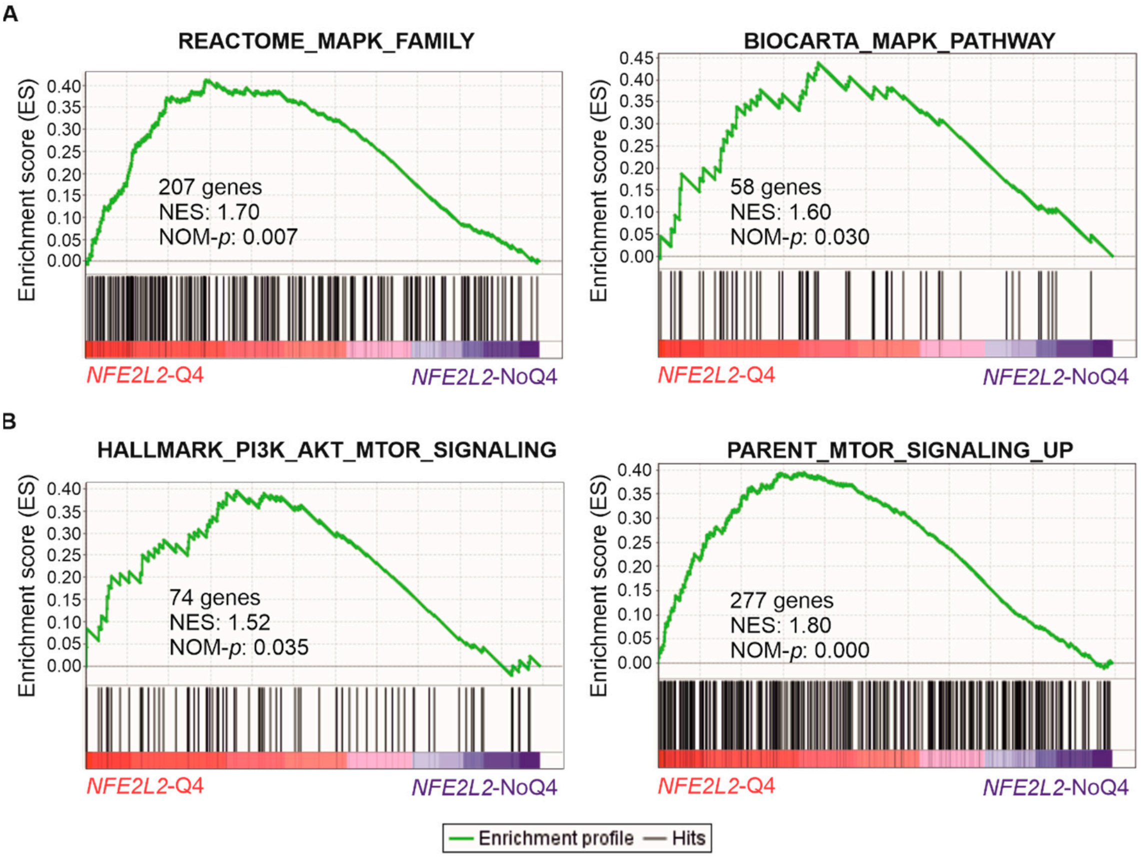

Supplementary Figure S2. Association between high *NFE2L2* levels and MAPK-ERK and PI3K-AKT-mTOR oncogenic signalling in T-ALL. Gene Set Enrichment Analysis was performed in 38 T-ALL patients with publicly available gene expression data [32], comparing patients belonging or not to the upper quartile of *NFE2L2* expression (*NFE2L2*-Q4 *vs* *NFE2L2*-NoQ4). **(A)** Two representative signatures for MAPK-ERK signalling pathway were selected from the Molecular Signatures Dabatase (MSigDB), and their systematic names are M27565 (REACTOME_MAPK_FAMILY_SIGNALING_CASCADES) and M13863 (BIOCARTA_MAPK_PATHWAY). **(B)** Two representative signatures for PI3K-AKT-mTOR signalling pathway were selected from the Molecular Signatures Dabatase (MSigDB), and their systematic names are M5923 (HALLMARK_PI3K_AKT_MTOR_SIGNALING) and M16909 (PARENT_MTOR_SIGNALING_UP). The green curve corresponds to the enrichment score (ES) curve, which is the running sum of the weighted score obtained with the GSEA software v4.2.1. NES, normalized enrichment score; NOM-*p*, nominal *p* value.

Supplementary Figure S3

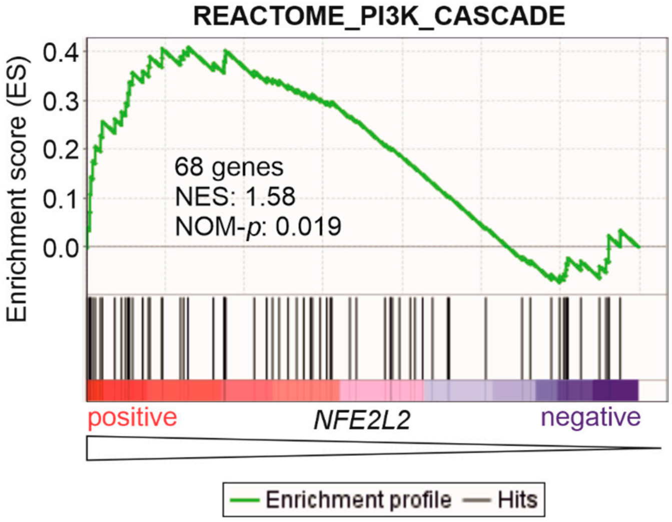

Supplementary Figure S3. Association of PI3K-AKT-MTOR pathway to T-ALL patients with high *NFE2L2* expression, in absence of *PTEN* mutations. Patients from the TARGET cohort not exhibiting loss-of-function *PTEN* mutations were selected (n=232) to perform Gene Set Enrichment Analysis (GSEA), based on their *NFE2L2* expression levels as a continuous variable. The REACTOME_PI3K_CASCADE signature (systematic name M16929) was selected from the Molecular Signatures Dabatase (MSigDB). The green curve corresponds to the enrichment score (ES) curve, which is the running sum of the weighted score obtained with the GSEA software v4.2.1. ROS, Reactive Oxygen Species; NES, normalized enrichment score; NOM-*p*, nominal *p* value.

Supplementary Figure S4

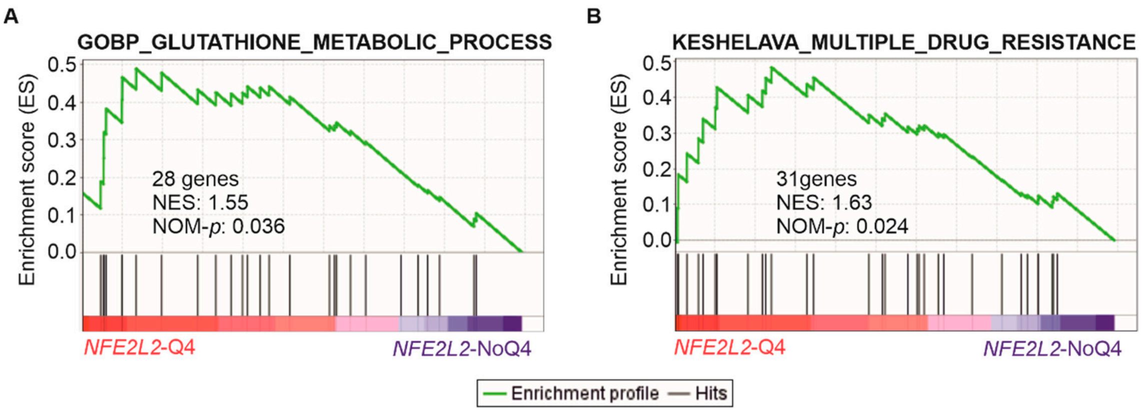

Supplementary Figure S4. Genetic signatures of glutathione metabolism and drug resistance in T-ALL patients with high *NFE2L2* expression. Gene Set Enrichment Analysis (GSEA) was performed in 38 T-ALL patients with publicly available gene expression data [32], comparing patients belonging or not to the upper quartile of *NFE2L2* expression (*NFE2L2*-Q4 *vs* *NFE2L2*-NoQ4). These signatures were selected from the Molecular Signatures Dabatase (MSigDB), and their systematic names are M14708 (GOBP_GLUTATHIONE_METABOLIC_PROCESS) and M12618 (KESHELAVA_MULTIPLE_DRUG_RESISTANCE). The green curve corresponds to the enrichment score (ES) curve, which is the running sum of the weighted score obtained with the GSEA software v4.2.1. NES, normalized enrichment score; NOM-*p*, nominal *p*-value.
